# The Adenoids but Not the Palatine Tonsils Serve as a Reservoir for Bacteria Associated with Secretory Otitis Media in Small Children

**DOI:** 10.1128/mSystems.00169-18

**Published:** 2019-02-12

**Authors:** Helena Fagö-Olsen, Laura Marie Dines, Christian Hjort Sørensen, Anders Jensen

**Affiliations:** a Department of Otolaryngology and audiology, Copenhagen University Hospital Rigshospitalet/Gentofte, Copenhagen, Denmark; b Department of Biomedicine, Faculty of Health Sciences, Aarhus University, Aarhus, Denmark; Teagasc Food Research Centre

**Keywords:** 16S rRNA genes, adenoids, adenotonsillectomy, microbiome, otitis media with effusion, tonsils

## Abstract

Acute otitis media (AOM), secretory otitis media (SOM), and acute pharyngotonsillitis are the most frequent reasons for visits to general practitioners, pediatricians, and otolaryngologists. Microbial colonization of the epithelial lining of Waldeyer’s lymphatic tissues, consisting of the palatine tonsils, lingual tonsils, adenoids, and Eustachian tube tonsil, is a well-known clinical challenge during infancy due to frequent episodes of upper respiratory tract infections. However, no previous studies have investigated the combined role of the palatine tonsils and the adenoids as a reservoir for pathogens associated with SOM in small children. We analyzed the combined crypt microbiome of the palatine tonsils and adenoids from 14 small children with hyperplasia of the tonsils or adenoids and 14 small children with SOM using 16S rRNA gene pyrosequencing. Our study demonstrated a significant difference between the microbiome of the adenoids and that of the palatine tonsils in the two groups but not between the two anatomical locations within the two groups. In particular, the potential pathogens Streptococcus pneumoniae, Haemophilus influenzae, and Moraxella catarrhalis were almost exclusively found in the adenoids of both patient groups, indicating that the adenoids and not the palatine tonsils are the main reservoir for potential pathogens leading to AOM and SOM.

**IMPORTANCE** Our findings that the microbiome differs between crypts of the adenoids and crypts of the palatine tonsils, including the relative abundances of potential pathogens such as Haemophilus influenzae, Streptococcus pneumoniae, and Moraxella catarrhalis, may be the stepping stone for further investigation of individual microbiomes in a longitudinal design that includes recording of the fluctuating health status of the child. Such studies may have the potential to lead to new preventive measurements such as implantation of protective nonpathogens at the nasopharynx as an alternative to adenoidectomy.

## Abstract (precis)

Our findings that the microbiome differs between crypts of the adenoids and crypts of the palatine tonsils, including the relative abundances of potential pathogens such as Haemophilus influenzae, Streptococcus pneumoniae, and Moraxella catarrhalis, may be the stepping stone for further investigation of individual microbiomes in a longitudinal design that includes recording of the fluctuating health status of the child. Such studies may have the potential to lead to new preventive measurements such as implantation of protective nonpathogens at the nasopharynx as an alternative to adenoidectomy.

## INTRODUCTION

The microbial colonization of the epithelial lining of Waldeyer’s lymphatic tissues, consisting of the palatine tonsils, lingual tonsils, adenoids, and Eustachian tube tonsil, is a well-known clinical challenge during infancy due to frequent episodes of upper respiratory tract infections. Acute otitis media (AOM), secretory otitis media (SOM), and acute pharyngotonsillitis are the most frequent reasons for visits to general practitioners, pediatricians, and otolaryngologists. Potential pathogens, such as Streptococcus pneumoniae, nonencapsulated Haemophilus influenzae, and Moraxella catarrhalis, have often been cultured from the nasopharynx and from middle-ear fluid of small children with AOM and with SOM (1–8). Furthermore, in culture-negative middle-ear aspirates, bacterial remnants of both Gram-negative and Gram-positive bacteria, including specific antibodies and antigen-antibody complexes, and activation of the complement system have been found, giving evidence of antecedent middle-ear involvement of certain bacteria (7, 9, 10). The quantitative nasopharyngeal load and diversity of potential pathogens differ according to the clinical and infectious status of the child (4, 11, 12), but other factors may also influence the composition of the microbiota, including the use of antibiotics, vaccination with relevant polysaccharides, and the interspecies interference of nonpathogens (5, 11, 13, 14). The more or less chronic inflammatory state within the nasopharyngeal mucosa, i.e., adenoiditis, coincides with hyperplasia (HP) of the lymphoid tissue with an increased number of germinal centers.

Adenoidectomy, tonsillectomy, and insertion of ventilating tubes into the tympanic membrane are frequent surgical procedures in small children with recurrent upper respiratory tract infections, SOM, and/or hyperplasia of the nasopharyngeal lymphoid tissue (11–13). In such children, results from prospective randomized controlled studies have shown long-term clinical effects of adenoidectomy and insertion of grommets by resolution of middle-ear effusion and, thereby, improvement of hearing. Tonsillectomy *per se* did not show any significant effect on middle-ear status (15–17). This may indicate differences in the composition of the microbiome of the different components of Waldeyer’s lymphoid tissues. Palatine tonsils are covered by stratified squamous epithelium, and the crypts are arranged in a lacunar manner, whereas the adenoids are covered by respiratory epithelium and the crypts are located in longitudinal folds. It seems reasonable to believe that these anatomical differences may play a role in the various forms of colonization of microorganisms within the Waldeyer’s lymphatic tissues.

Previously, we showed by 16S rRNA gene sequence analysis that the microbiomes of the palatine tonsils in children with chronic tonsillitis and in children with hyperplasia of the palatine tonsils do not differ in overall composition in the individual. They may (rarely) harbor species like S. pneumoniae and H. influenzae, which are potential pathogens for the nasopharynx and middle-ear cavity (18). Other studies of the microbiome of different loci within the Waldeyer´s ring by 16S rRNA gene analysis showed that, in particular, the adenoids, but also other parts of the nasopharynx, may be a reservoir for pathogens responsible for AOM and SOM in young children (19–23). However, no previous studies have investigated the combined roles of the palatine tonsils and the adenoids as reservoirs for pathogens associated with SOM in small children.

In this study, we examined, at the species level by means of 16S rRNA gene pyrosequencing, the crypt microbiomes of the palatine tonsils and adenoids in each child in two groups of small children. The goal was to provide a thorough description of the complex microbiome of these two important sections of the Waldeyer’s ring in relation to small children with SOM and/or hyperplasia of the adenoids.

## RESULTS

**FIG 1:**
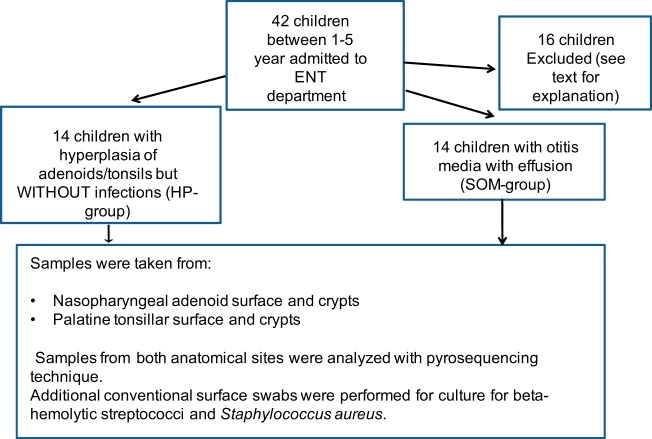
Schematic presentation of the sample collection.

In total, 168 samples from 44 children were collected. Samples from 8 children were ruined during transportation, and samples from 7 children could not be analyzed because of technical problems or because they, after sampling, were found not to fit the inclusion criteria. Samples from one child were excluded because she developed symptoms of chickenpox immediately after the operation (Fig. 1).

Finally, 112 samples from 28 children (14 children in the HP group and 14 children in the SOM group; Table 1) were eligible for analysis.

**TABLE 1:** Overview of the included patients

Patient ID^*a*^	Group	Age (mo)	Gender	Content of middle ear
1	HP	42	M	Air
3	HP	65	M	Air
6	HP	41	F	Air
8	HP	42	F	Air
11	HP	36	M	Air
13	HP	40	F	Air
14	HP	38	F	Air
15	HP	42	F	Air
16	HP	31	F	Air
17	HP	42	M	Air
18	HP	24	M	Air
19	HP	42	M	Air
20	HP	35	M	Air
22	HP	63	M	Air
12	SOM	19	F	Fluid
21	SOM	35	F	Fluid
23	SOM	59	M	Fluid
25	SOM	17	M	Fluid
26	SOM	29	M	Fluid
28	SOM	27	F	Fluid
33	SOM	15	M	Fluid
35	SOM	21	F	Fluid
39	SOM	53	F	Fluid
40	SOM	42	M	Fluid
42	SOM	21	F	Fluid
43	SOM	34	F	Fluid
45	SOM	28	F	Fluid
46	SOM	24	M	Fluid

a ID, identifier.

After our rigorous error filtering of the reads, the number of sequences ranged from 52,366 derived from the adenoid sample from patient 17 to 5,083 from the tonsil sample from patient 25 (see Table S1 in the supplemental material) corresponding, to 57% to 73% of the raw data. No significant differences in the percentage of sequences retained in each sample after processing between the two sequencing sites were observed. Although the numbers of reads from the two sequencing sites differed, the sequencing coverage was very high in all samples (Table S1). To eliminate any biases caused by the sequencing depth, though, we normalized all our samples to 5,083 sequences per sample before any downstream analysis was performed. The mean length of the processed sequences was 567 bases, thereby covering the entire V1-V3 region of the 16S rRNA gene.

10.1128/mSystems.00169-18.3 TABLE S1 Sample characteristics after reduction of sequencing errors. Download Table S1, DOCX file, 0.02 MB. Copyright © 2019 Fagö-Olsen et al. 2019 Fagö-Olsen et al. This content is distributed under the terms of the Creative Commons Attribution 4.0 International license.

**FIG 2:**
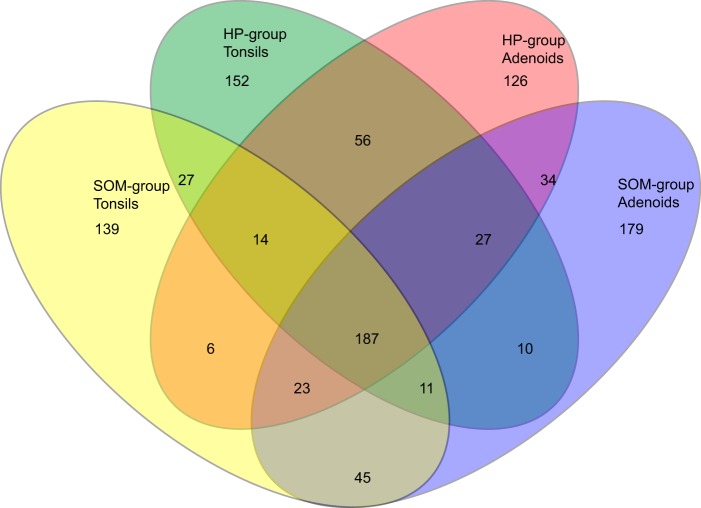
Venn diagram showing overlap of observed OTUs for the four groups clustered at 98.5% similarity. A total of 187 OTUs were found in at least one sample from each of the four groups. A total of 1,036 OTUs were found across all 56 samples.

**FIG 3:**
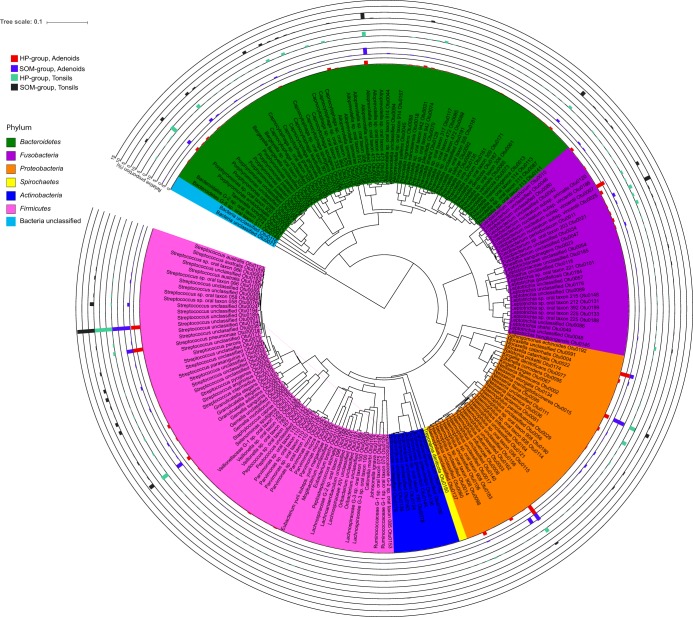
Unweighted pair group method using average linkages (UPGMA) tree showing the phylogenetic relationship of the 200 most abundant OTUs across all samples. The tree file was generated using representative sequences from all 200 OTUs in MEGA 7 and visualized using iToL software. Each phylum is color highlighted, and the average percent abundancies of each OTU for the four groups are shown.

**FIG 4:**
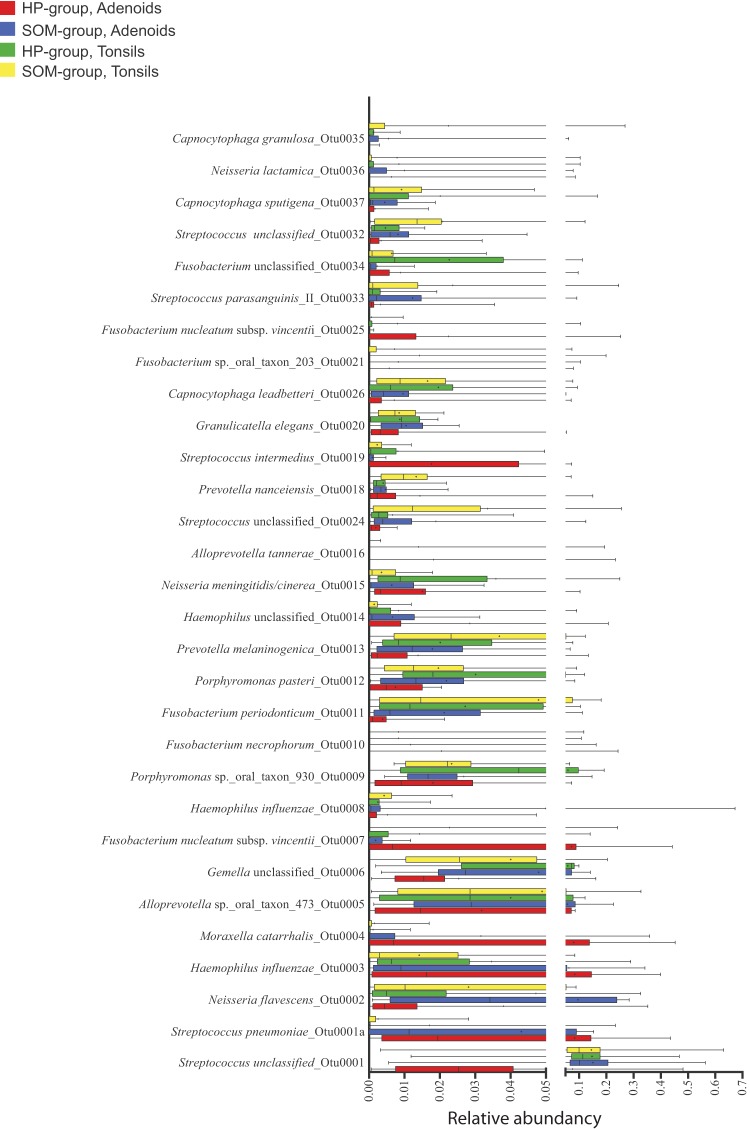
Box plot of the 30 most abundant OTUs. Whiskers show the range of the relative abundancies of each OTU in the four groups. Median and average values are shown as vertical lines and a cross, respectively.

The number of operational taxonomic units (OTUs) at 98.5% sequence similarity ranged from 44 OTUs in the adenoid sample from patient 1 in the HP group to 136 OTUs in the adenoid sample from patient 42 in the SOM group. The total number of OTUs observed across all samples was 1,036, but only the following five OTUs were found in all 56 samples: OTU0001 (*Streptococcus* unclassified, but BLAST analysis confirmed that the OTU belonged to the S. mitis/*oralis*/*infantis* cluster); OTU0002 (Neisseria flavescens); OTU0006 (*Gemella* unclassified); OTU9 (*Porphyromonas* oral taxon 930); and OTU0013 (Prevotella melaninogenica) (Table S2). Dividing the samples anatomically added two more ubiquitous OTUs, each corresponding to a site as follows: OTU0011 (Fusobacterium periodonticum [tonsils] and OTU0020 (Granulicatella elegans [adenoids]). In contrast, 18% (187 OTUs) were found in at least one of the samples from the four groups (Fig. 2). The total number of OTUs observed at the 98.5% sequence similarity level ranged from 452 in the tonsils of the SOM group to 516 in the adenoids of the SOM group (Fig. 2). The 10 most abundant OTUs accounted for 46.6% of the total sequences ranging from 8.4% in the tonsils of patient 22 from the HP group to 91.9% in the adenoids of patient 16 from the HP group (Table S2). A phylogenetic tree of the 200 most abundant OTUs with the average abundance of the OTUs for each of the four groups shows that the most abundant OTUs were found in the *Bacteroidetes*, *Fusobacteria*, *Proteobacteria*, and *Firmicutes* phyla, with the *Firmicutes* phylum containing most (64) of the 200 most abundant OTUs (Fig. 3). The most abundant OTU (OTU0001) accounted for 7.7% (adenoids of the HP group) to 15.1% (adenoids of the SOM group) of the total amount of sequences in each group. Similarly for all of the most abundant OTUs, however, the intersample variation of the abundancy was high (Fig. 4).

10.1128/mSystems.00169-18.4 TABLE S2 Taxonomic classification of OTUs and the relative abundances (%) in each sample. Core OTUs are marked in blue (all), green (tonsils), or red (adenoids) (Table S3). Download Table S2, XLSX file, 0.3 MB. Copyright © 2019 Fagö-Olsen et al. 2019 Fagö-Olsen et al. This content is distributed under the terms of the Creative Commons Attribution 4.0 International license.

The results of alpha diversity analysis of comparisons of the four groups showed that the number of OTUs found in the adenoids from the HP group was significantly lower than the number found in adenoids from SOM group (see Fig. S1a in the supplemental material). Similarly, the Invsimpson and the npshannon diversity index values were significantly lower for the samples from the adenoids of the HP group than for those from the other three groups (Fig. S1b and c).

10.1128/mSystems.00169-18.1 FIG S1 Microbial richness and diversity based on the number of observed OTUs (A), the Invsimpson diversity index (B), and the npshannon diversity index (C) in each of the four groups. Student’s *t* test was used to identify differences between the groups (*, <0.05; **, <0.01). Download FIG S1, EPS file, 0.7 MB. Copyright © 2019 Fagö-Olsen et al. 2019 Fagö-Olsen et al. This content is distributed under the terms of the Creative Commons Attribution 4.0 International license.

**FIG 5:**
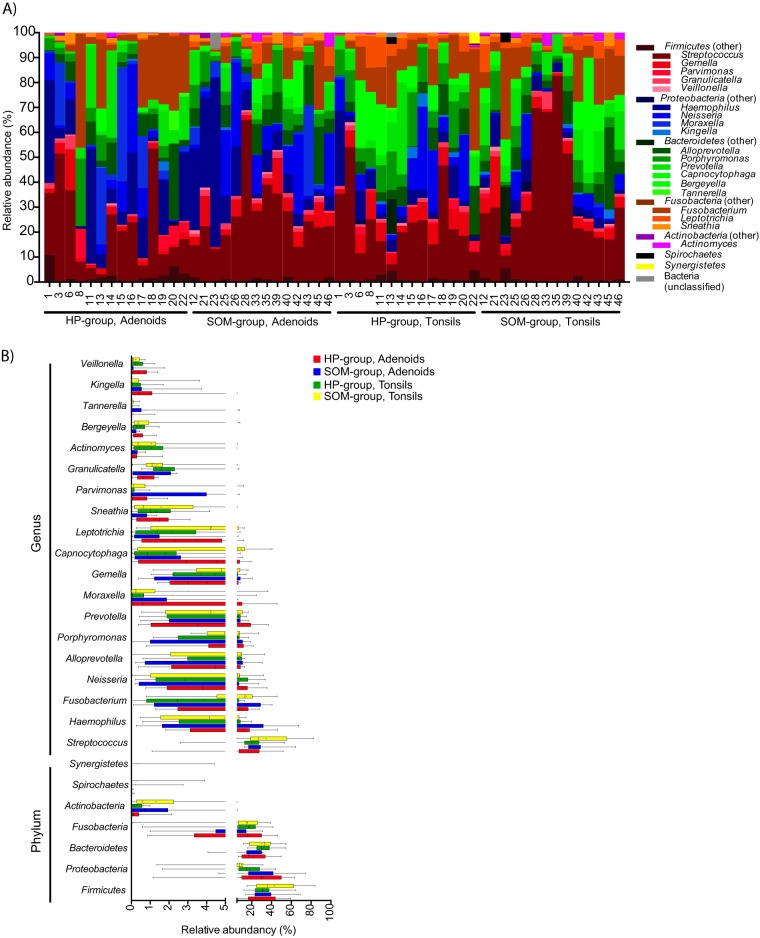
Bar plot (A) and box plot (B) of the relative abundances of the phyla found and the 20 most abundant genera in each of the four groups. Median and average values are shown as vertical lines and crosses, respectively, in panel B.

Taxonomic assignment of the sequences revealed that seven different phyla were detected across all our samples (Fig. 5). In addition, some sequences could not be assigned to any known phyla (e.g., 6.6% of the sequences from the adenoids of sample 23 of the SOM group). The most abundant phylum in all four groups was either *Firmicutes* (range, 28.9% in adenoids of the HP group to 42.7% in tonsils of the SOM group) or *Proteobacteria* (range, 9.5% in tonsils of the SOM group to 31.9% in adenoids of the HP group) (Fig. 5). Only four phyla (*Firmicutes*, *Proteobacteria*, *Fusobacteria*, and *Bacteroidetes*) were found in all of our samples, and, in general, high intersample variation at the phylum level was observed (Table S3). A total of 81 different genera were detected across all the samples (Table S3), with the 20 most abundant genera accounting for an average of 97.3% of all the sequences (range, 88.5% in adenoids from sample 1 of the HP group to 99.8% in tonsils from sample 16 of the HP group). On average, 28 different genera were found per sample, with no differences among the four groups (range, 26 genera in the tonsils of HP group to 30 genera in adenoids from SOM group). *Streptococcus* was the most abundant genus (average, 25.6%) followed by *Fusobacterium* (average, 11.1%) and *Haemophilus* (10.3%), and similarly to the results determined at the phylum level, intersample variability was very high (Fig. 5b). Using the Human Oral Microbiome Database (HOMD) as the reference for taxonomic assignment of the sequences, we were able to assign the sequences to the species level and a total of 164 validly described species were detected. In addition, 103 oral taxa that are considered distinct but that are not validly described as species were detected (Table S3). The genera that included most species and oral taxa were *Prevotella*, with 30 species and oral taxa, and *Streptococcus*, with 19 species and oral taxa. The most abundant validly described species were Haemophilus influenzae (average, 10.0%), Fusobacterium nucleatum (average, 6.4%) and Neisseria flavescens (average, 6.2%), but, similarly to the observations at higher taxonomic rankings, intersample variability was significant (Table S3).

10.1128/mSystems.00169-18.5 TABLE S3 Raw output of the taxonomic classification of sequences using HOMD. Download Table S3, XLSX file, 0.3 MB. Copyright © 2019 Fagö-Olsen et al. 2019 Fagö-Olsen et al. This content is distributed under the terms of the Creative Commons Attribution 4.0 International license.

**FIG 6:**
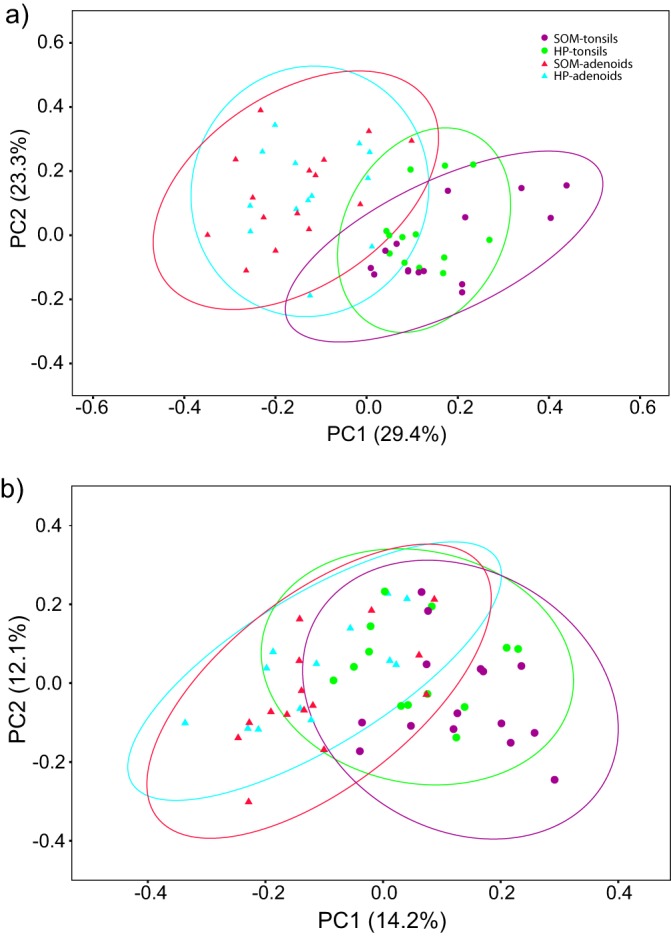
PCoA plots comparing the bacterial communities from the four groups using weighted UniFrac distances (A) and the OTU structure (thetaYC calculator) using 98.5% sequence similarity for clustering (B). The percentage of variation explained by each principal coordinate (PC) is indicated on the axes. Each point represents a microbial community. Weighted UniFrac distances from the phylogenetic tree generated from the Clearcut program implemented in the Mothur software. Confidence ellipses (95%) are shown for each group.

To compare the beta-diversity data from our four groups, we generated principal-component analysis (PCoA) plots of the weighted UniFrac distances of the phylogenetic tree generated from the Clearcut program and the thetaYC distances of the OTUs at a 98.5% similarity level (Fig. 6). In the PCoA plots, differences in the overall structure between the four different groups were not clearly visible and permutational multivariate analysis of variance (PERMANOVA) confirmed that no significant differences in the overall bacterial community structure were present between the adenoid samples from the SOM or the HP group (*P* = 0.17) and the tonsils from the SOM or the HP group (*P* = 0.23) using weighted UniFrac distances (Table S4). However, PERMANOVA of the thetaYC distances revealed borderline significance (*P* = 0.046) for the results of comparisons of the bacterial structures in the adenoids of the SOM group and the HP group but no difference between the bacterial community structures of the tonsil samples from the SOM group and HP group (*P* = 0.091). The bacterial community structures, however, were significantly different between the adenoid and tonsil samples both in comparing all the samples (PERMANOVA *P* = 0.005 [weighted UniFrac] and *P* = 0.007 [thetaYC]) and in comparing samples only within the same group (SOM or HP). In contrast, we observed a tendency toward positive correlation of the relative abundancies of some of the 15 most abundant OTU between the tonsil and adenoid samples, including Streptococcus_unclassified (OTU1), Neisseria flavescens (OTU2), and Alloprevotella sp. oral taxon 473 (OTU5) (Fig. S2). However, no clear correlation was observed for the potential pathogens S. pneumoniae (OTU1a), H. influenzae (OTU3 and OTU8), and M. catarrhalis (OTU4) (Fig. S2).

10.1128/mSystems.00169-18.2 FIG S2 Correlation between the relative proportions of the 15 most abundant OTUs in the tonsils and adenoids. Linear regression analysis showed that the abundances of some OTUs were positively correlated between the anatomical sites. Download FIG S2, EPS file, 1.2 MB. Copyright © 2019 Fagö-Olsen et al. 2019 Fagö-Olsen et al. This content is distributed under the terms of the Creative Commons Attribution 4.0 International license.

10.1128/mSystems.00169-18.6 TABLE S4 PERMANOVA comparing different patient groups. Download Table S4, DOCX file, 0.01 MB. Copyright © 2019 Fagö-Olsen et al. 2019 Fagö-Olsen et al. This content is distributed under the terms of the Creative Commons Attribution 4.0 International license.

**FIG 7:**
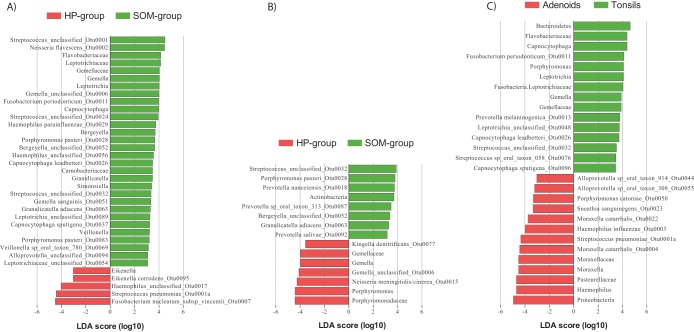
Differentially abundant bacterial taxa (phyla, families, genera, and OTUs) identified by linear discriminant analysis (LDA) coupled with effect size measurements (LEfSe) in comparisons of the HP group and the SOM group from the tonsils (A) and the adenoids (B) and from the two body sites (C). Only taxa that met the significant linear discriminant analysis threshold value of 3.5 are shown.

**FIG 8:**
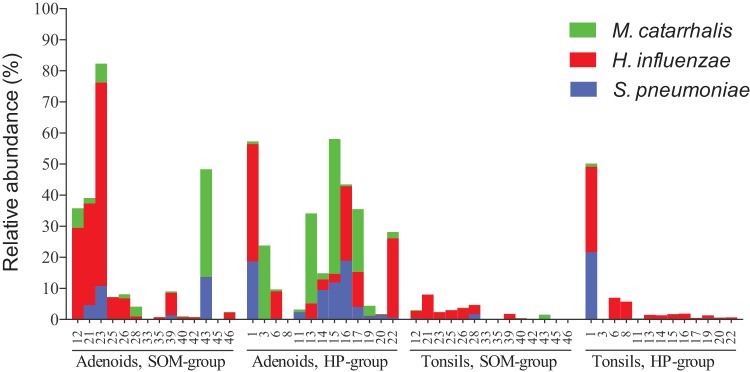
Relative abundances of the three species M. catarrhalis, H. influenzae, and S. pneumoniae in each of the 56 samples grouped according to the group definition.

To detect which taxa (phyla, family, genera, OTUs) were responsible for the observed differences in the microbial composition between the adenoid samples and tonsil samples, we used linear discriminant analysis (LDA) coupled with the linear discriminant analysis effect size (LEfSe) algorithm (Fig. 7). In agreement with our PCoA analysis and the PERMANOVA testing, our LEfSe analysis detected only a few taxa that were differentially abundant between the tonsil samples from the SOM group and those from the HP group (Fig. 7A). Most of the differentially abundant OTUs were low-abundancy OTUs, although the phylum *Actinobacteria* was significantly more abundant in the tonsils from the SOM group. Similar tendencies were detected in comparing the adenoid samples from the SOM and HP groups, although the number of taxa that were differentially abundant between the two groups was slightly higher (Fig. 7B). In addition, 8 of the 30 most abundant OTUs were significantly more abundant in one of the two groups, supporting the idea of borderline significance found by the PERMANOVA of the thetaYC distances between the two groups. Interestingly, S. pneumoniae (OTU0001a) was significantly more abundant in the adenoids of HP group than in the adenoids of SOM group. Fusobacterium nucleatum subsp. *vincentii* (OTU0007) was abundant in many of the samples from the adenoids of the HP group but was virtually absent from the adenoid samples from the SOM group. LeFSe analysis confirmed the differential abundancy of this OTU between the two groups. Unlike the tonsil samples, no phyla were differentially abundant between the groups of adenoids. Comparing the two different anatomical locations (adenoids and the tonsils), all three species typically associated with otitis media, H. influenzae, S. pneumoniae, and M. catarrhalis, were significantly more abundant in the adenoids than in the tonsils (Fig. 7C) and, with the exception of the tonsils of patient 1 of the HP group, were rarely present in the tonsils of those in either the SOM or the HP group (Fig. 8). The high abundancy of *Haemophilus* and *Moraxella* in the adenoids accounted for the increased proportions of the *Proteobacteria* phylum on the adenoids. The increased proportion of *Bacteroidetes* in the tonsil samples was mostly due to the *Porphyromonas* and *Capnocytophaga* genera. In addition, the anaerobic genus *Leptotrichia* was more abundant in the tonsil samples; overall, however, anaerobic bacteria were not more abundant in the tonsil samples than in the adenoid samples.

Beta-hemolytic streptococci associated with tonsillitis and, to a lesser degree, otitis media, i.e., Streptococcus pyogenes (Lancefield group A *Streptococcus*) and Streptococcus dysgalactiae subsp. *equisimilis* (Lancefield group C or G *Streptococcus*), were either present in very few samples at a low abundancy (with the exception of the tonsils from patient 39, where S. pyogenes accounted for 31.2% of the total sequences) or not present at all, as was the case for S. dysgalactiae subsp. *equisimilis* (Table S3). Similar, Fusobacterium necrophorum, which recently has been linked to tonsillitis in teenagers and young adults and to otitis media in children (24, 25), was found in only seven samples. When present, the abundancy was mostly high in both of the paired samples, with the highest proportion in the tonsil samples (between 4.5% and 24.3% in six of the samples). Another important pathogen, Staphylococcus aureus, was detected rarely and in only seven samples at a very low abundancy (below 0.3%).

Cultivation of the samples for beta-hemolytic streptococci and S. aureus showed that 10 samples from six patients were positive for S. pyogenes whereas S. dysgalactiae subsp. *equisimilis* was absent (Table S5). All culture-positive samples were also positive by sequencing, and both samples for which sequences classified as S. pyogenes accounted for more than 1% of the total (tonsils of patient 1, 3.2%; tonsils of patient 39, 31.2%) were positive by culture. S. aureus was found in two samples, both of which were positive by sequencing as well (Table S5).

10.1128/mSystems.00169-18.7 TABLE S5 Growth of beta-hemolytic streptococci and S. aureus from tonsil and adenoid samples. Download Table S5, DOCX file, 0.01 MB. Copyright © 2019 Fagö-Olsen et al. 2019 Fagö-Olsen et al. This content is distributed under the terms of the Creative Commons Attribution 4.0 International license.

## DISCUSSION

To our knowledge, this was the first study to collectively characterize the microbiome of the palatine tonsillar crypts and adenoid crypts in small children with SOM and/or adenoid hyperplasia. We found that the bacterial community was relatively diverse and dominated by species belonging to the genera *Streptococcus*, *Haemophilus*, and *Fusobacterium*. The diversity of the core microbiome was low at the OTU level, suggesting high interpersonal variability, but it also may reflect our stringent cutoff of 98.5% similarity. On the other hand, by using this stringent OTU cutoff, the OTUs may reflect the biological impact of the presence of phylogenetically different clones of the same taxon more precisely. This is exemplified by the presence of several different OTUs of H. influenzae in general as well as within individual samples. The presence of multiple clones of H. influenzae, especially in aboriginal children, had been observed previously and has been linked to development disease in the nasopharynx, including otitis media (26, 27). However, previous investigations have shown that the multiple clones are present in healthy children as well (4, 28).

We found that the overall bacterial community structures differed between crypts of the adenoids and the palatine tonsils. This is in contrast to most of the relatively few other studies that have compared the bacteriology of the adenoids and palatine tonsils in children by culture. Brook and Shah (29) found overall similarity in the bacterial species isolated from the surfaces of the adenoids and palatine tonsils of children suffering from recurrent adenotonsillitis. Likewise, Taylan et al. (30) also found similarity in the bacteria sampled from the surfaces of palatine tonsils and adenoids of children suffering from recurrent tonsillitis and/or obstructive symptoms. Conceivably, the different results obtained in our study were due to the higher resolution of culture-independent sequencing techniques. In addition, culture may favor fast-growing bacteria, which again may contribute to more-similar bacterial population results than the less biased DNA-based method. Furthermore, there may be differences in the composition of the microbiota of the surface and crypts of the lymphoepithelial tissues. Surface samples from the nasopharynx and oropharynx may easily be contaminated by saliva, tears, and other secretions. The use of the transnasal swab technique for sampling the nasopharyngeal microflora may result in an overestimation of the occurrence of S. aureus (21, 31).

Only one other study investigated the relation between the bacterial populations in the adenoids and palatine tonsils by culture-independent techniques (23). Liu et al. (23) found that the bacterial communities of adenoids and palatine tonsils in one single 8-year-old boy suffering from chronic SOM overlapped to some degree but also found clear differences in the bacterial composition. We also found overlap between the adenoids and palatine tonsils in the bacterial composition of the samples despite the overall dissimilarities. Furthermore, we found that the proportions of some of the most abundant OTUs were positively correlated within the adenoids and palatine tonsils from the same patient. These findings clearly demonstrate that, in spite of the overall differences between the anatomical sites with respect to their bacterial composition, several bacterial species were found to be occasionally residing in both anatomical locations. Interestingly, this was not the case for the classical pathogens S. pneumoniae, H. influenzae, and M. catarrhalis. These species were almost completely absent from the palatine tonsils of the children. This may explain why tonsillectomy *per se* did not seem to have any therapeutic effect on SOM in small children. Thus, palatine tonsils may not serve as a reservoir for the adenoids and middle-ear cavity for these potentially pathogenic bacteria. The variabilities in the overall community structure (including potential pathogens) is most likely explained by differences in the surface epithelia and the gross anatomical structures of the crypts of the two lymphoid tissue examined. Our samples were collected at a time with no acute infection. The overlap in the bacterial species residing at the two anatomical locations, including S. pneumoniae, M. catarrhalis, and H. influenzae, may be greater in the presence of an active infection.

S. pneumoniae, H. influenzae, and M. catarrhalis often co-occurred in the adenoid samples, indicating either that they displayed some kind of symbiotic relationship or that they preferred the same environmental conditions present in the adenoid crypts of our patient groups but without experiencing any major competition. Virtually similar findings have been observed in nasopharyngeal aspirates and swabs from small children (32, 33). It has been suggested that interactions between S. pneumoniae and M. catarrhalis may occur because the latter acts as an indirect pathogen by producing beta-lactamase, facilitating growth of S. pneumoniae in the presence of beta-lactam antibiotics (34, 35).

We did not detect any differences between the two patient groups in the overall bacterial composition of the palatine tonsil and adenoid samples. This is, to our knowledge, the first study to have compared the bacterial compositions of these two locations in children with hyperplasia and/or SOM using DNA sequence-based technology. Our findings clearly support the assumption that hyperplasic adenoids represent the result of an immunological response to potential pathogenic bacteria colonizing the adenoids (36). Small children with hyperplastic adenoids breathe almost exclusively through the mouth, resulting in hyperplastic palatine tonsils and, occasionally, in colonization by bacteria otherwise associated with the colonization of the nasopharynx and middle-ear cavity.

F. necrophorum has been associated with pharyngotonsillitis in adolescents and young adults, and recent studies also associated F. necrophorum with otitis media in young children (24, 37). We found F. necrophorum in high proportions in three patients in samples of both the palatine tonsils and the adenoids. Our results indicate that F. necrophorum may be able to colonize the adenoids through an initial colonization of the palatine tonsils, subsequently causing otitis media in those patients. However, further studies are needed to determine the role of F. necrophorum in the upper respiratory tract of young children.

S. aureus is considered a potential pathogen in otitis media and pharyngotonsillitis and was previously demonstrated to be present in the adenoids and palatine tonsils of children suffering from recurrent acute otitis media and SOM by both culture and culture-independent techniques (22, 38). Interestingly, staphylococci, including S. aureus, were shown to be almost completely absent from our samples both by sequencing and by culture. These results are congruent with our previous study investigating the bacteriology of the tonsils in children and adults (18). Using the same protocol for DNA extraction and sequencing as was used in this study, we have analyzed several samples from the nose of young children (data not published). The proportion of S. aureus was high in those samples, suggesting that the low rate of detection of S. aureus by sequencing in this study was not due to methodological problems. Combining those results with our findings by culture, we consider the data indicating the low level and quantity of S. aureus to be valid. A possible explanation could be that S. aureus is present mostly on the surface epithelium of the palatine tonsils and adenoids but not in the crypts from which our samples were taken. However, a study by Nistico et al. (39) showed that S. aureus was present both on the surface and in the crypts of the adenoids from children with chronic otitis media with perforation of the tympanic membrane and thereby had access to the flora of the external ear canal. Although our findings are in contrast to those obtained most other studies, they are similar to those in recent studies where S. aureus was found in very low proportion in samples from the adenoids of children with SOM both by specific PCR (40) and by MiSeq sequencing (21). Furthermore, the role of S. aureus in naso- and oropharyngeal diseases such as pharyngotonsillitis and otitis media may be overestimated due to the bacterium’s easy and rapid growth in culture on blood agar and its characteristic large colonies, which are hardly likely to be overlooked on inspection. However, given the relatively small number of samples examined in this study, such differences should always be verified with a larger sample size. Similar assumptions might be true for beta-hemolytic streptococci. However, we found S. pyogenes in more samples than S. aureus both by sequencing and culture, and S. pyogenes was found most often in both the samples of the palatine tonsils and the adenoids from the same child. As with our findings with respect to F. necrophorum, this might suggest that the colonization of the tonsils with S. pyogenes in children may lead to the colonization of the adenoids and may eventually may cause otitis media in some children.

In recent studies, the species Alloiococcus otitidis has been associated with otitis media in children, being found in high proportions in middle-ear effusion from children with SOM by 16S rRNA sequencing (19–21). The same studies also found a high prevalence of the bacterium on the surface of the adenoids but at a very low proportion (0.01%). This genus was completely absent in both our palatine tonsil samples and adenoid samples. The absence in our samples might have been due to the fact that A. otitidis is an obligate aerobe and not able to grow well under the conditions present in the crypts of the palatine tonsils and adenoids.

In conclusion, this was, to our knowledge, the first study to use pyrosequencing techniques to examine the microbiome of the crypts of the palatine tonsils and adenoids in children suffering from SOM and/or hyperplasia of the adenoids. Our study results demonstrate significant differences between the microbiome of the adenoids and that of the palatine tonsils. We believe that those differences were due to the different surface epithelia and the distinct anatomical characteristics of the lymphoid tissue in these loci. We are also fully aware of the possible confounding influence of environmental factors on the nasopharyngeal microbial milieu. However, we choose not to record such factors except the inclusion and exclusion criteria, as it would be almost impossible to analyze the impact of these confounders due to the relatively low number of children who were included in the study. To our knowledge, prior infections, such as catarrhalia and acute and recurrent OM, and antibiotic consumption are important events influencing the composition of the nasopharyngeal flora. The significance of other confounding environmental variables such as breastfeeding and parental smoking have not yet been fully clarified. S. pneumoniae, H. influenzae, and M. catarrhalis often co-occurred in the adenoids in both our SOM and HP groups. They are known to be potential pathogens but may also be detected in the normal microbiome of children without infectious symptoms. Therefore, it is likely that the individual compositions of the pediatric upper airway microbiome represent a critical factor that may either potentiate or protect against infection by respiratory pathogens. In this context, it is interesting that the recurrent loads of potential pathogens and mitogens have been suggested to negatively influence the immune regulation and termination of local B cells in the adenoids and middle-ear mucosa (9, 41–43).

Further investigation of individual microbiomes in a longitudinal design that includes recording of the fluctuating health status of the child may lead to new preventive measures such as implantation of protecting nonpathogens into the nasopharynx as an alternative to adenoidectomy.

## MATERIALS AND METHODS

### Ethics statement.

The study protocol was approved by The Danish Scientific Ethics Committee (reference 1-10-72-100-13), and written informed consent was obtained from parents of the children included in the study.

### Subjects.

Children between 1 and 5 years of age were consecutively included if referred for surgery at the Ear-Nose-Throat (ENT) Department at Rigshospitalet/University Hospital of Copenhagen, Denmark, or in one private ENT clinic for 1 year, 2012 to 2013, except the months of April and May. Exclusion criteria included treatment with antibiotics within 1 month prior to surgery, immunosuppressive therapy, or cleft palate treatment and inability to obtain informed consent in either Danish or English. The referred parents filled out a questionnaire and were interviewed by the ENT specialist concerning previous episodes of upper respiratory tract infections that included antibiotic treatment, obstructive symptoms such as snoring, breathing and eating problems and sleep apnea, and hearing problems. At the time of sampling, none of the included children had an acute infection. SOM was defined as accumulation of middle-ear fluid verified by tympanometry, paracentesis, and clinical evaluation by an ENT specialist no more than 3 months prior to surgery.

Two groups of children were included in the study (Fig. 1; see also Table 1). (i) The hyperplasia group (HP group) included patients who had experienced adenoidectomy or adenotonsillectomy due to significant hyperplasia of the lymphoid tissues of the palatine tonsils and/or adenoids causing obstructive symptoms such as snoring, breathing and eating problems, and sleep apnea but without prior AOM or SOM during the previous year. (ii) The SOM group included patients who had been subjected to insertion of ventilation tubes into the tympanic membrane and to adenoidectomy due to hearing problems and who had had recurrent episodes of upper respiratory tract infections, including catarrhalia. The Danish guidelines for referral to surgery with insertion of ventilating tubes indicate that there must be symptoms of SOM and require three separate measurements of fluid in middle ear with at least one month between measurements. Five children in this group had previously experienced a few episodes of otitis media treated by antibiotics, but none of the children could be classified as prone to otitis.

### Sample collection.

Samples were collected during general anesthesia before surgery commenced. Four samples were collected from each patient, including two samples from both palatine tonsils and two samples from the adenoids. One sample from each anatomical location was used for pyrosequencing and one for conventional bacterial culture. For pyrosequencing, samples from both the tonsillar and adenoid crypts collected bilaterally were later pooled to one sample for each anatomical location. Thereafter, a conventional swabbing procedure was performed for the culture analysis. Great care was taken to avoid touching the outer surface of the tonsil or other parts of the oral and pharyngeal mucosa. In collecting samples from the adenoids, the soft palate was carefully elevated and retracted to obtain full visualization before sampling from the depth of the adenoid crypts was performed. The samples intended for pyrosequencing were placed in a 1.5-ml Eppendorf tube with 750 µl of PowerSoil DNA kit bead solution (Mo-Bio, Carlsbad, CA), and swabs for conventional culture were placed in Stewart’s transport medium (SSI, Denmark). Samples were sent to the Department of Biomedicine at Aarhus University on dry ice (DNA samples only), where all further analyses were performed.

### DNA extraction.

Upon arrival of the samples at Aarhus University, DNA was immediately extracted using a PowerLyzer PowerSoil DNA isolation kit (Mo-Bio, Carlsbad, CA) and the manufacturer’s instructions with the exception that a FastPrep FP120 cell disruptor (Thermo Savant) was used for the release of DNA. After thawing the samples, the 750 µl of PowerSoil DNA kit bead solution and the cotton swab were twice subjected to brief vortex mixing for 60 s each time to ensure that the bacteria were suspended. The suspension, together with 60 µl of PowerSoil C1 solution, was then added to a PowerLyzer glass bead tube. DNA was released from the cells using a FastPrep FP120 cell disruptor (Thermo Savant) at 5.5 ms^−1^ for 30 s. The treatment was repeated three times, and the samples were cooled on ice between the treatments. After the bead beating step, the instructions in the user manual was followed from paragraph 7. DNA was eluted in 100 µl of the C6 buffer supplied with the kit.

### PCR and pyrosequencing.

Partial 16S rRNA gene sequences were amplified from the samples using the barcoded-primer approach to multiplex pyrosequencing. Using the 27F primer (5′-Fusion A adapter-barcode-AGAGTTTGATCCTGGCTCAG-3) and the 926R primer (5′-Fusion B adapter-CCGTCAATTCMTTTRAGT-3′), a DNA fragment spanning the V1-V5 region of the 16S rRNA gene was amplified by PCR. The first part of the 16S rRNA is generally considered the most optimal region for distinguishing oral and nasal bacteria, and the use of that part also allows us to differentiate Streptococcus pneumoniae from other species of the mitis group of streptococci on the basis of the specific sequence signature of S. pneumoniae at position 203 (44). Experiments using the PCR mixtures were carried out in a total volume of 25 µl, comprising 10 µl of 1:10-diluted DNA sample, 2.5 µl of 10× PfuUltra II reaction buffer (Stratagene), a 200 nM concentration of each primer (IDT), 0.5 µl of PfuUltra II fusion HS DNA polymerase (Stratagene), 1 µl deoxynucleoside triphosphate (dNTP) mix (25 mM concentration of each dNTP), and 9 µl of molecular-biology-grade water. PCR was performed using the following cycle conditions: an initial denaturation step at 95°C for 2 min followed by 30 cycles of denaturation at 95°C for 20 s, annealing at 55°C for 20 s, and elongation at 72°C for 15 s and then a final elongation step at 72°C for 3 min. Three PCRs were performed for all samples, and all PCR products were verified on a 1% agarose gel. The correct bands were then excised from the gel, pooled, and purified using a NucleoSpin extract kit (Macherey-Nagel). The concentration of the purified PCR products was measured on a NanoDrop 2000 spectrophotometer (Thermo Scientific) as well as on a 1% agarose gel. The samples from the adenoids and the palatine tonsils, respectively, were then pooled in equal amounts and sequenced unidirectionally from the forward primer end by the use of GS FLX+ technology at the Department of Genomic and Applied Microbiology, Institute of Microbiology and Genetics, University of Göttingen, Göttingen, Germany.

DNA extraction controls were included as well as positive and negative controls for PCRs. Because the negative extraction controls showed no PCR products, they were not included for sequencing.

### Sequence processing and analysis.

The open-source, platform-independent software program mothur v.1.39.5 (http://www.mothur.org) was used to process and analyze the pyrosequencing data (45). Processing of raw pyrosequencing reads was done using Schloss Standard Operating Procedure (SOP) 454 for pyrosequencing reads (http://www.mothur.org/wiki/454_SOP) (46) with some modifications. Briefly, sequencing noise was initially reduced using the shhh.flows commands, which represent an implementation of the PyroNoise algorithm in the mothur software package (47). Sequences containing more than two mismatches to the forward primer sequence, containing one mismatch to the barcode sequence, containing more than eight homopolymers, or containing any ambiguous characters were removed before denoising of the sequences was performed. The maximum number of flows was set at 1,150 as recommended by Patrick Schloss (personal communication). After denoising, sequences were aligned using Silva reference alignment version 128 and the resulting alignment was then filtered such that all of our sequences overlapped only at identical regions. To further reduce sequencing errors, a preclustering step implementing a pseudo-single-linkage algorithm originally developed by Huse et al. (48) was performed with the diff-value set to 4. Chimeras were removed using the build-in version of the UCHIME algorithm in mothur (49). Lastly, all singletons were removed before analysis to preclude inclusion of sequences representing potential contamination or of sequence errors that had not been detected in the previous steps. All samples were rarefied to 5.083 sequences per sample prior to performing downstream analyses using the Sub.sample command in mothur. We choose to rarefy our samples, as this normalization method is still one of the best available, especially in cases in which the library sizes differ considerably between samples as shown by Weiss et al. (50). All sequences that passed the quality control in each sample were used for classification together with the Bayesian method and taxonomic outline ver. 14.51 from the Human Oral Microbiome Database (HOMD) (http://www.homd.org/) (51). The confidence cutoff was set to 80%. HOMD is a curated database of known oral bacterial species and allows one to classify sequences to the species level. Additionally, to confirm the species identification of the sequences found with the HOMD database, we also used the method and the species-specific 16S sequence signatures as described previously (44) for the 20 most abundant genera and 50 most abundant species found with HOMD. As only very minor differences were found in the classification, we used the classification found by HOMD throughout the study. One exception, however, was that HOMD identified 15,907 sequences as Neisseria meningitidis. On further inspection, it was obvious that these sequences were also similar to those of N. cinerea, which is not in HOMD. Therefore, we denoted these sequences as N. meningitidis/N. cinerea as these two species cannot be distinguished using the V1-V3 region of the 16S rRNA gene. Furthermore, all sequences identified as unclassified *Streptococcus* were screened by BLAST analysis to determine whether they belonged to the mitis group of streptococci. All sequences that belonged to this group, as well as the sequences already identified as representing species belonging to the mitis group of streptococci by HOMD, were screened for the presence of the S. pneumoniae-specific sequence signature at position 203 in the 16S rRNA gene; S. pneumoniae is the only *Streptococcus* species that harbors a cysteine at that location (44). All sequences with this nucleotide were subtracted from the classification and pooled as S. pneumoniae.

Preliminary sorting of the sequences was done by clustering the sequences into operational taxonomic units (OTU) defined by a 98.5% similarity level using the opticlust clustering method. The 98.5% similarity level was chosen because many named oral bacterial species have high sequence identity in their 16S rRNA genes, particularly among *Streptococcus*, *Actinomyces*, *Haemophilus*, and *Neisseria*. Therefore, a number of different species are likely to be combined into the same OTU in applying the more commonly used distance of 0.03, as previously discussed by others (52, 53). The resulting OTUs were then classified using the classify.otu command in mothur and the HOMD database. Again, OTUs classified as N. meningitidis were reassigned as N. meningitidis/N. cinerea. Ultimately, all sequences from the OTUs found to belong to the mitis group of Streptococci using the described methods were again screened for the presence of the S. pneumoniae-specific sequence signature in the 16S rRNA gene. These sequences were subtracted from the original OTUs and pooled into a new OTU resembling S. pneumoniae and named OTU0001a. Invisimpson and Shannon diversity indices were calculated in mothur, and Student’s paired and unpaired *t* tests were used to detect differences between the groups in Invisimpson and Shannon diversity indices. Differences were considered significant at *P* < 0.05. Linear discriminant analysis (LDA) coupled with effect size measurement (LEfSe) was used to detect bacterial taxa (phyla, genera, and species) that were differentially abundant between groups. The alpha values for the factorial Kruskal-Wallis and pairwise Wilcoxon tests were set to 0.05, and the LDA score threshold for discriminative features was set to 3.5. The online version of the LEfSe program (https://huttenhower.sph.harvard.edu/galaxy/) was used (56). Principal-component analysis (PCoA) plots based on Unweighted Unifrac distances generated from a phylogenetic tree produced in the Clearcut program (54) and on thetaYC distances of the OTUs clustering at a 98.5% similarity level were visualized in R ver. 3.4.2. To test if the separation of the defined groups visualized by the principal-component analysis (PCoA) plots was statistically significant, a PERMANOVA test was performed using the Adonis function in the vegan package (version 2.4 to 4) implemented in R with 999 permutations. Differences were considered significant at *P* < 0.05. Interactive Tree of Life (iToL) software (http://itol.embl.de) (55) was used to visualize the phylogenetic relationships and abundancy of the 200 most abundant OTUs.

Correlational analysis was performed using linear regression analysis with Prism6 software. A *P* value cutoff of 0.01 was used to determine the significance of the slope from nonzero.

### Cultivation.

Swabs for cultivation were plated on 5% blood agar (SSI, Denmark) and incubated at 37°C for 24 h in a CO_2_-enriched atmosphere to enable detection of the presence of Staphylococcus aureus and beta-hemolytic streptococci (Streptococcus pyogenes and S. dysgalactiae subsp. *equisimilis*). After incubation, suspected colonies were picked and identified using matrix-assisted laser desorption ionization–time of flight MALDI-TOF (Bruker).

### Data availability.

All raw reads were deposited in the NCBI Sequence Read Archive (SRA) with accession number PRJNA422760.
