# Supplementary material for: The Adenoids but Not the Palatine Tonsils Serve as a Reservoir for Bacteria Associated with Secretory Otitis Media in Small Children
Source: mSystems. 2019 Feb 12;4(1):e00169-18. doi: 10.1128/mSystems.00169-18 (PMC6372837; doi:10.1128/mSystems.00169-18)
Supplement: TABLE S1 [file mSystems.00169-18-st001.docx]

| Table S1 | | | | | | |
| --- | --- | --- | --- | --- | --- | --- |
| Patient | Bodysite | Passed filtered reads | Good's coverage | OTUs (98.5%) | invsimpson diversity index | npshannon diversity index |
| 1 | Adenoid | 43,721 | 0.998 | 44 | 4.41 | 2.07 |
| 1 | Tonsil | 11,579 | 0.997 | 76 | 5.68 | 2.38 |
| 3 | Adenoid | 35,590 | 0.996 | 85 | 3.73 | 2.14 |
| 3 | Tonsil | 7,663 | 0.998 | 105 | 4.15 | 2.50 |
| 6 | Adenoid | 18,352 | 0.996 | 68 | 5.90 | 2.42 |
| 6 | Tonsil | 5,301 | 0.999 | 70 | 13.44 | 3.09 |
| 8 | Adenoid | 25,752 | 0.998 | 46 | 3.98 | 1.89 |
| 8 | Tonsil | 7,009 | 0.999 | 72 | 14.17 | 3.10 |
| 11 | Adenoid | 41,515 | 0.997 | 57 | 6.47 | 2.36 |
| 11 | Tonsil | 9,644 | 0.998 | 89 | 17.30 | 3.28 |
| 12 | Adenoid | 49,427 | 0.995 | 128 | 8.37 | 3.15 |
| 12 | Tonsil | 7,161 | 0.998 | 133 | 22.88 | 3.76 |
| 13 | Adenoid | 40,944 | 0.997 | 81 | 6.68 | 2.54 |
| 13 | Tonsil | 9,803 | 1.000 | 96 | 19.95 | 3.48 |
| 14 | Adenoid | 40,550 | 0.996 | 120 | 16.34 | 3.43 |
| 14 | Tonsil | 8,291 | 0.998 | 98 | 10.60 | 3.08 |
| 15 | Adenoid | 28,230 | 0.995 | 104 | 4.07 | 2.31 |
| 15 | Tonsil | 10,670 | 0.998 | 90 | 10.52 | 2.96 |
| 16 | Adenoid | 5,448 | 0.997 | 66 | 4.19 | 1.90 |
| 16 | Tonsil | 11,385 | 0.999 | 55 | 5.52 | 2.29 |
| 17 | Adenoid | 52,366 | 0.996 | 76 | 7.32 | 2.52 |
| 17 | Tonsil | 13,359 | 0.997 | 86 | 8.84 | 2.70 |
| 18 | Adenoid | 42,343 | 0.995 | 86 | 3.36 | 1.98 |
| 18 | Tonsil | 9,291 | 0.998 | 89 | 6.24 | 2.76 |
| 19 | Adenoid | 40,346 | 0.997 | 83 | 8.19 | 2.82 |
| 19 | Tonsil | 8,499 | 0.998 | 78 | 12.90 | 3.06 |
| 20 | Adenoid | 51,632 | 0.997 | 70 | 7.31 | 2.54 |
| 20 | Tonsil | 7,415 | 0.997 | 102 | 9.85 | 3.06 |
| 21 | Adenoid | 11,605 | 0.997 | 72 | 5.58 | 2.40 |
| 21 | Tonsil | 10,765 | 0.999 | 84 | 8.51 | 2.82 |
| 22 | Adenoid | 38,580 | 0.996 | 103 | 10.22 | 3.11 |
| 22 | Tonsil | 12,059 | 0.998 | 103 | 10.59 | 3.15 |
| 23 | Adenoid | 5,235 | 0.995 | 84 | 2.12 | 1.46 |
| 23 | Tonsil | 13,201 | 0.999 | 79 | 8.66 | 2.84 |
| 25 | Adenoid | 5,083 | 0.996 | 118 | 12.47 | 3.37 |
| 25 | Tonsil | 9,008 | 0.998 | 99 | 22.11 | 3.57 |
| 26 | Adenoid | 5,193 | 0.996 | 91 | 7.23 | 2.72 |
| 26 | Tonsil | 8,157 | 0.997 | 106 | 11.73 | 3.15 |
| 28 | Adenoid | 46,944 | 0.998 | 98 | 3.08 | 2.36 |
| 28 | Tonsil | 9,905 | 0.997 | 92 | 2.27 | 1.86 |
| 33 | Adenoid | 28,322 | 0.999 | 101 | 18.63 | 3.53 |
| 33 | Tonsil | 8,099 | 0.998 | 71 | 8.78 | 2.82 |
| 35 | Adenoid | 30,505 | 0.998 | 120 | 20.34 | 3.58 |
| 35 | Tonsil | 9,475 | 0.999 | 46 | 6.16 | 2.27 |
| 39 | Adenoid | 35,969 | 0.997 | 76 | 6.09 | 2.44 |
| 39 | Tonsil | 10,373 | 0.998 | 85 | 7.98 | 2.94 |
| 40 | Adenoid | 50,496 | 0.996 | 117 | 19.67 | 3.50 |
| 40 | Tonsil | 13,403 | 0.998 | 96 | 16.33 | 3.40 |
| 42 | Adenoid | 53,625 | 0.994 | 136 | 9.86 | 3.29 |
| 42 | Tonsil | 8,189 | 0.998 | 96 | 10.91 | 3.28 |
| 43 | Adenoid | 49,804 | 0.995 | 85 | 4.76 | 2.21 |
| 43 | Tonsil | 12,466 | 0.997 | 91 | 14.36 | 3.27 |
| 45 | Adenoid | 43,100 | 0.996 | 81 | 6.88 | 2.41 |
| 45 | Tonsil | 6,596 | 0.998 | 71 | 6.30 | 2.52 |
| 46 | Adenoid | 46,114 | 0.995 | 111 | 10.61 | 3.17 |
| 46 | Tonsil | 7,665 | 0.997 | 107 | 16.45 | 3.39 |
