# Supplementary material for: The Adenoids but Not the Palatine Tonsils Serve as a Reservoir for Bacteria Associated with Secretory Otitis Media in Small Children
Source: mSystems. 2019 Feb 12;4(1):e00169-18. doi: 10.1128/mSystems.00169-18 (PMC6372837; doi:10.1128/mSystems.00169-18)
Supplement: TABLE S4 [file mSystems.00169-18-st004.docx]

| **Table S4**. | | | |
| --- | --- | --- | --- |
| Group 1 | Group 2 | Weighted Unifrac | thetaYC |
| SOM-group (adenoids) | HP-group (adenoids) | 0.17 | 0.046 |
| SOM-group (tonsils) | HP-group (tonsils) | 0.23 | 0.091 |
| Tonsils (all) | Adenoids (all) | 0.005 | 0.007 |
| Tonsils (SOM-group) | Adenoids (SOM-group) | 0.008 | 0.013 |
| Tonsils (HP-group) | Adenoids (HP-group) | 0.003 | 0.004 |
