# Supplementary material for: The Adenoids but Not the Palatine Tonsils Serve as a Reservoir for Bacteria Associated with Secretory Otitis Media in Small Children
Source: mSystems. 2019 Feb 12;4(1):e00169-18. doi: 10.1128/mSystems.00169-18 (PMC6372837; doi:10.1128/mSystems.00169-18)
Supplement: TABLE S5 [file mSystems.00169-18-st005.docx]

| **Table S5**. | | | | | | |
| --- | --- | --- | --- | --- | --- | --- |
| Patient no. | Group | Body site | *S. aureus* | *S. pyogenes* | *S. equisimilis* |  |
| 1 | Hp-group | Tonsils | ­ | + |  |  |
| 3 | Hp-group | adenoids |  | + |  |  |
| 3 | Hp-group | Tonsils |  | + |  |  |
| 6 | Hp-group | Tonsils |  | + |  |  |
| 14 | Hp-group | adenoids |  | + |  |  |
| 14 | Hp-group | Tonsils |  | + |  |  |
| 16 | Hp-group | Tonsils | + |  |  |  |
| 17 | Hp-group | adenoids |  | + |  |  |
| 17 | Hp-group | Tonsils |  | + |  |  |
| 39 | SOM-group | Tonsils |  | + |  |  |
| 39 | SOM-group | adenoids |  | + |  |  |
| 40 | SOM-group | Tonsils | + |  |  |  |
